# Explainable blind image quality assessment with closed-loop semantic guidance and distortion diagnosis

**DOI:** 10.1038/s41598-026-51187-6

**Published:** 2026-05-09

**Authors:** Chenye Song, Fujiang Yuan, Zhiwang Zhang

**Affiliations:** 1 School of Computer Science and Technology, Taiyuan Normal University, Jinzhong, 030619 Shanxi China; 2 School of Computer Science and Data Engineering, NingboTech University, Ningbo, 315000 Zhejiang People’s Republic of China

**Keywords:** Blind image quality assessment, Decision support, Vision language models, Distortion diagnosis, Adaptive intelligent systems, Engineering, Mathematics and computing

## Abstract

In large-scale visual systems, particularly in automated image triage and preprocessing pipelines, the reliability of downstream computer vision tasks depends on the quality of perceptual inputs and the interpretability of assessment outcomes. Existing blind image quality assessment methods are typically formulated as offline black-box predictors that produce a single scalar score. While indicating degradation, it provides limited actionable guidance, semantic awareness, and insufficient diagnostic information for system-level decision-making. To address these limitations, this work proposes an interpretable blind image quality assessment framework designed to serve as a perceptual monitoring component that provides diagnostic support for automated visual systems. The approach incorporates high-level semantic priors from a frozen vision–language model into a Vision Transformer-based assessment stream via feature-wise linear modulation, enabling content-aware quality evaluation. In parallel, a distortion diagnosis branch is jointly optimized to identify dominant degradation types and generate structured diagnostic cues that support adaptive restoration. Experiments on standard benchmarks demonstrate strong consistency with human subjective judgments, achieving Spearman’s rank correlation coefficients of 0.9509 on TID2013 and 0.9408 on KADID-10k. The model also operates at 65 frames per second, indicating a favorable balance between accuracy, interpretability, and efficiency.

**Supplementary information:**

is available for this paper at 10.1038/s41598-026-51187-6.

## Introduction

With the widespread deployment of vision-based artificial intelligence^1^, particularly in cloud-based visual analysis and high-throughput image repositories, automated systems are increasingly required to process large volumes of unconstrained visual data. In such pipelines, images are frequently affected by diverse algorithmic, physical, and transmission-induced degradations. Within these systems, perception, reasoning, and decision-making are tightly coupled in a closed-loop manner, such that system reliability depends on the continuous assessment of perceptual inputs during operation. These integrated perception—reasoning pipelines are increasingly embedded in agent architectures^2^, where degraded perceptual inputs may adversely influence reasoning outcomes and subsequent behaviors. Prior studies indicate that system performance and safety are closely associated with the robustness of visual perception under quality degradation^3,4^, particularly in safety-critical applications^5^.

Image Quality Assessment (IQA) has been extensively investigated as a means of quantifying visual degradation. In recent years, learning-based approaches have demonstrated strong consistency with human subjective judgments on standard benchmark datasets^6–9^. However, most existing IQA methods are primarily designed for offline evaluation and typically produce a single scalar quality score. This conventional paradigm presents two main limitations. First, traditional IQA models generally lack explicit semantic awareness. As a result, it becomes difficult to distinguish degradations in task-relevant regions from those in less critical areas. Second, a scalar score provides limited actionable guidance for subsequent processing. This is because it does not provide a structured characterization of the underlying distortion types.

**Fig. 1:**
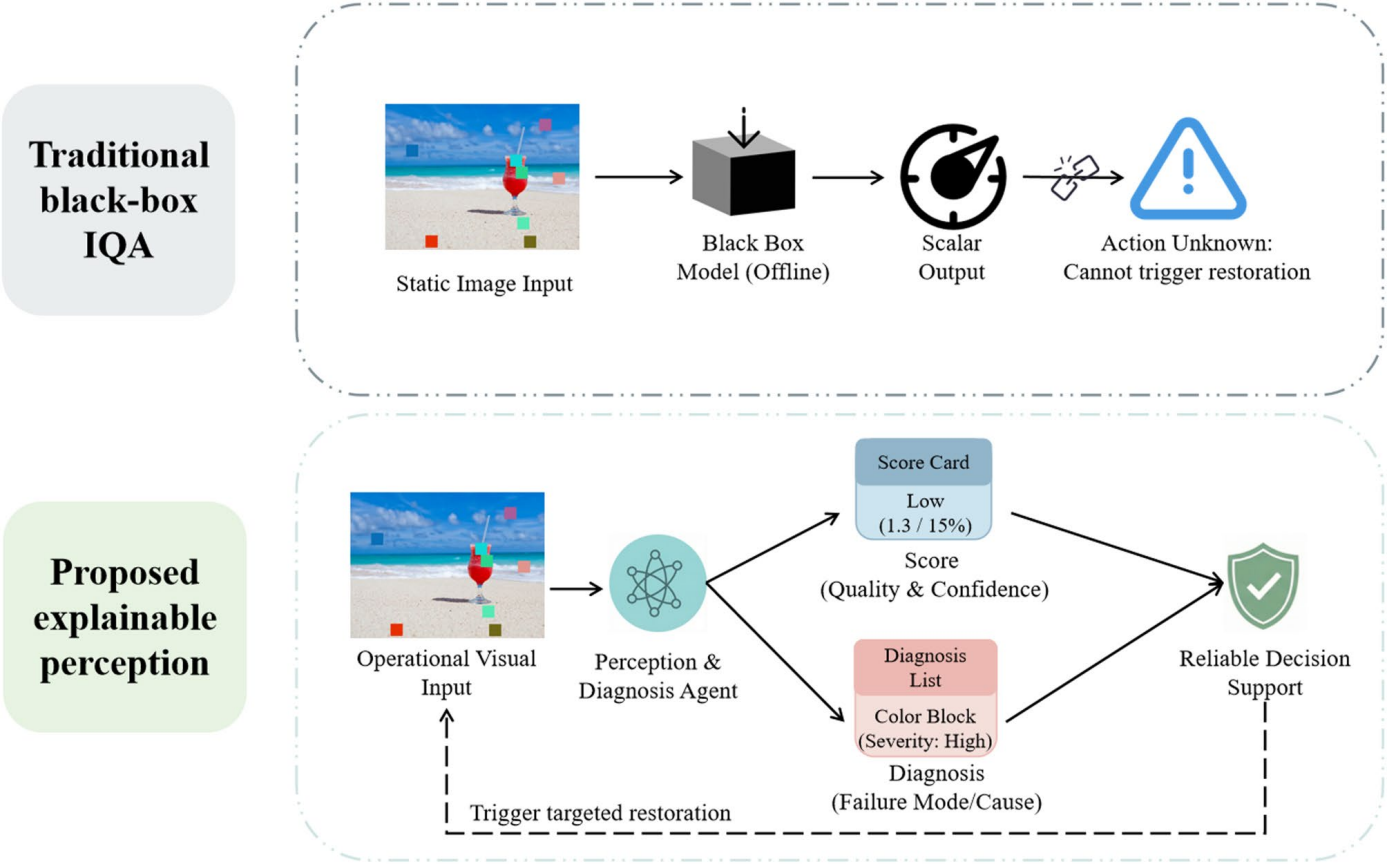
Framework shift from black-box quality scoring to explainable perception support.

To illustrate this conceptual shift, Fig. 1 contrasts the conventional offline IQA paradigm with the proposed diagnostic-driven decision-support perspective. Rather than functioning as open-loop evaluators, the proposed framework repositions IQA as an interpretable diagnostic perception component embedded within the operational loop. It performs real-time analysis of incoming visual inputs and outputs both quality estimates and structured diagnostic signals. For example, when degradation is detected, the diagnosis branch generates structured indicators, such as identifying JPEG2000 compression or Gaussian noise. These signals can guide downstream processing modules to activate corresponding restoration mechanisms, including deblocking filters or targeted denoising networks, thereby enabling automated self-repair.

Under this formulation, IQA is reframed from a conventional quality quantification tool into a functional component within an adaptive perception pipeline. To explicitly distinguish our fundamental technical contributions from existing paradigms, this work introduces three key methodological shifts. First, prior CLIP-based IQA models often rely on computationally intensive cross-modal attention, which risks disrupting pre-trained visual priors. To address this issue, we introduce a zero-initialized Feature-wise Linear Modulation FiLM strategy for lightweight and non-destructive semantic injection. Second, in conventional multi-task blind image quality assessment (BIQA), distortion classification serves merely as an auxiliary output. In contrast, our approach repurposes the diagnostic loss as an explicit structural regularizer. This mechanism encourages the initially entangled visual features to evolve into more physically interpretable and linearly separable sub-spaces within a single forward pass. Third, generic semantic or diagnostic enhancement methods typically focus on descriptive analysis. In contrast, our framework generates actionable pre-routing signals to trigger targeted downstream image restoration. This mechanism effectively shifts IQA from a passive scoring tool to an active system-level monitor.

The main contributions of this work are summarized as follows.

1. A diagnostic-driven BIQA framework is introduced to provide perception-level assurance for automated visual pipelines. The method reformulates BIQA from a passive scoring task into an interpretable monitor that generates actionable diagnostic signals to inform monitoring–diagnosis–restoration workflows. This capability facilitates adaptive visual processing in dynamic environments.
2. A semantic-aware injection mechanism is developed to enhance monitoring reliability, where high-level semantic priors from a frozen vision–language model are incorporated via a zero-initialized feature-wise linear modulation strategy. Compared to computationally intensive cross-modal attention, this design enables a lightweight and non-destructive integration of semantic information without disrupting pre-trained visual priors. Consequently, this design improves sensitivity to degradations in semantically important regions and enhances alignment with human perception.
3. An explicit distortion diagnosis branch is integrated to facilitate adaptive restoration. Beyond serving as an auxiliary classifier, the multi-task architecture also acts as a structural regularization mechanism, encouraging the shared feature space to be organized into more interpretable and semantically meaningful subspaces. The resulting diagnostic signals provide structured guidance for downstream restoration modules within a perception–action feedback loop.
4. The operational feasibility and robustness of the proposed approach are validated through extensive experiments, demonstrating a favorable balance between perceptual accuracy and inference efficiency for online deployment, while zero-shot evaluations indicate competitive generalization across previously unseen distortion scenarios in open-world environments.

## Related work

While notable progress has been achieved in perceptual quality modeling, existing research remains distributed across three largely independent dimensions, including model-centric performance optimization, semantic enhancement through foundation models, and system-level reliability mechanisms. The absence of a unified perspective that jointly considers perceptual accuracy, semantic interpretability, diagnostic capability, and operational efficiency limits the effective deployment of IQA as a reliable monitoring component in closed-loop systems. In the following subsections, representative developments in these related domains, namely blind image quality assessment, foundation model-based perceptual modeling, and reliability assurance for intelligent systems, are reviewed. Through a comparative analysis of their respective strengths and limitations, the algorithmic and operational context underlying the proposed diagnostic-driven framework is established.

### Blind image quality assessment

Early BIQA methods primarily relied on convolutional neural networks (CNNs) to learn quality-sensitive representations from distorted images. These approaches demonstrated strong capability in modeling low-level degradation patterns such as noise and blur; however, their reliance on local receptive fields inherently constrained the modeling of long-range dependencies and global perceptual context.

To overcome this limitation, Transformer-based BIQA models have been widely investigated in recent years. Representative studies adopt Vision Transformer backbones and attention mechanisms to facilitate global–local feature interaction, yielding improved robustness under complex and heterogeneous distortion conditions^10,11^. Subsequent works further enhanced performance by introducing adaptive attention designs, hierarchical regression strategies, and self-supervised or large-scale pretraining schemes to improve structural modeling and data efficiency^12,13^. In addition, biologically inspired architectures and multi-task learning frameworks have been explored to improve sensitivity to perceptually salient regions^14^.

Although these advances have substantially improved BIQA accuracy, most existing methods are primarily optimized for offline quality prediction and focus on low-level visual statistics. As a result, their ability to capture high-level semantic degradation and to provide diagnostic signals suitable for system-level adaptation remains relatively underexplored, motivating the need for perceptual assessment frameworks that extend beyond scalar quality regression.

### Foundation models for perceptual quality assessment

To alleviate the limited semantic awareness of conventional BIQA models, recent studies have explored the integration of vision–language foundation models into perceptual quality assessment. By leveraging semantic priors learned from large-scale image–text data, these models offer a promising pathway toward enriching perceptual representations beyond purely visual distortion cues.

Recent efforts have incorporated CLIP-based representations into IQA pipelines through prompt learning, quality-aware token modeling, and multi-stage training strategies^15,16^. Further improvements have been achieved by enhancing cross-modal interaction via multi-scale attention mechanisms and semantic attribute modeling, enabling more semantically informed quality inference^17^. Other works have focused on stabilizing prediction through decoupled training schemes or two-stage optimization strategies^18^. In addition, interactive assessment systems have been proposed to extend IQA outputs from scalar scores to textual descriptions, improving interpretability in domain-specific applications such as medical imaging^19^.

Despite these advances, most foundation model-based approaches primarily exploit semantic priors for passive score regression or descriptive analysis, while the modeling of machine-actionable diagnostic cues remains relatively underexplored. Furthermore, existing CLIP-enhanced IQA methods often rely on computationally intensive cross-modal attention, which may disrupt pre-trained visual priors. In contrast, our approach introduces a zero-initialized, non-destructive semantic modulation strategy that reduces computational overhead and avoids multi-stage or heavily interactive designs. This highlights the importance of lightweight architectures that can achieve effective feature disentanglement while maintaining a balance between semantic awareness and operational efficiency.

### Perception reliability and decision support in intelligent systems

Beyond algorithmic accuracy, operational reliability has become a critical concern in high-stakes automated visual pipelines, including medical image analysis and large-scale multimedia content moderation. Due to the high-dimensional input space and complex decision processes of deep neural networks, exhaustive offline testing is insufficient to guarantee safe operation under all conditions, making continuous monitoring an essential component of system assurance. Recent studies have explored AI-assisted monitoring mechanisms and reliability-aware algorithms for distributed intelligent systems^20,21^. Model-driven approaches have also been proposed to support continuous specification and monitoring of operational properties^22^.

However, existing assurance mechanisms predominantly focus on decision logic consistency or high-level behavioral constraints, while perceptual data quality at the system front end has received comparatively limited attention. In real-world deployments, image degradation caused by sensor noise, motion blur, or transmission artifacts is common and can propagate through perception pipelines, potentially affecting downstream decisions. Although data-centric AI studies emphasize handling noisy and corrupted data throughout the system lifecycle^23^, most solutions rely on offline data processing and are not designed for dynamic operational environments.

In self-adaptive systems, effective planning and execution largely depend on reliable monitoring signals. Recent advances have improved adaptive decision-making through generative AI-assisted MAPE-K loops^24^ and online reinforcement learning-based configuration strategies^25^. Nevertheless, the effectiveness of such mechanisms ultimately hinges on the availability of perceptual monitoring components that can jointly assess input quality and provide interpretable diagnostic feedback for adaptive processing.

### Image restoration and quality-driven routing

A critical downstream application of BIQA is to guide image and video restoration. Recent advances in this field have yielded specialized architectures tailored to distinct degradation types. For example, the Deep Dense Multi-Scale Network (DDMSNet)^26^ incorporates semantic and depth priors to achieve state-of-the-art image desnowing performance. For video restoration tasks in the temporal domain, the Enhanced Spatio-Temporal Interaction Network (ESTINet)^27^ is proposed with an efficient spatio-temporal interaction design for high-performance video deraining. To address dynamic motion blur, the authors of^28^ developed DBLRNet, which employs 3D convolutions to model complex spatio-temporal blurring processes; this network is further integrated into a generative adversarial framework (DBLRGAN) via adversarial learning to enhance the visual realism of restoration results. In addition, the Multi-Branch Linear Transformer Expanded by Taylor Formula (MB-TaylorFormer V2)^29^ is designed as a lightweight, efficient architecture that delivers exceptional multi-task restoration capabilities.

Despite these promising developments, the efficacy of restoration algorithms is intrinsically linked to reliable quality assessment. State-of-the-art restoration networks exhibit strong domain specificity and rely heavily on precise degradation priors. As systematically validated by the Multi-Cause Blur (MC-Blur) benchmark^30^—in which models are evaluated across diverse degradation types, including high-frame-rate motion blur, defocus blur, large-kernel ultra-high-definition (UHD) motion blur, and real-world mixed blur—even top-performing models suffer from severe performance degradation and fail to generalize when applied to mismatched degradations.

This key limitation underscores the critical necessity of our proposed diagnostic IQA framework. Blindly applying degradation-specialized restoration networks to unmatched inputs often introduces severe secondary artifacts. By explicitly diagnosing the underlying degradation causes in real time, our framework provides essential pre-routing signals to enable optimal selection of restoration strategies. This design ensures that advanced restoration algorithms are activated exclusively under their optimal operating conditions, bridging the gap between conventional passive quality assessment and adaptive, system-level active image restoration.

## Methods

In this section, we present the proposed explainable semantic-aware BIQA framework. Designed to enhance perception reliability and decision support for intelligent systems, the method jointly addresses quality estimation and degradation reasoning within a unified architecture. The detailed methodology is structured into four key components: Dual-Stream Semantic Injection, Global-Local Feature Interaction, Distortion Diagnosis Branch, and Multi-Task Prediction and Joint Optimization. The specific design and optimization strategy of each component are introduced in the following subsections.

### Overall architecture

**Fig. 2:**
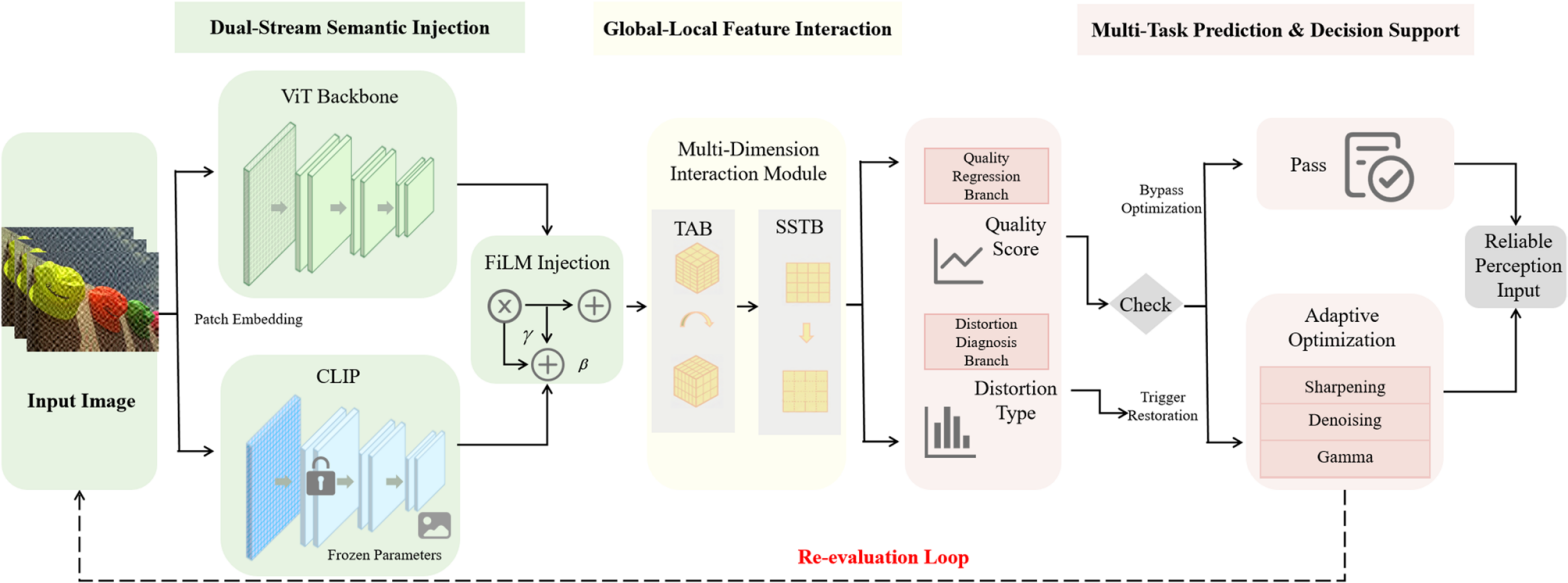
Overall architecture of the proposed closed-loop perception framework.

As illustrated in Fig. 2, the proposed framework integrates semantic-aware perception into a Closed-Loop Perception Framework. While the visual pipeline is structurally organized into three macro-modules, the methodology follows four logical stages:

Dual-stream semantic injection: A frozen CLIP encoder extracts high-level semantic priors, which are injected into the ViT backbone via FiLM. This ensures the quality assessment is explicitly guided by semantic context.

Global-local feature interaction: We employ a Multi-Dimension Interaction Module that consists of Transposed Attention Blocks (TAB) and Scale Swin Transformer Blocks (SSTB)^10^. This design robustly captures both global channel dependencies and local spatial distortions, reinforced by the injected semantics.

Distortion diagnosis branch: To enable interpretability, a dedicated parallel branch is nested within the prediction head to explicitly classify dominant distortion types. This separates specific degradation features from the generic quality regression task.

Multi-task prediction and joint optimization: Finally, the Multi-Task Prediction Head unifies these tasks to drive the Decision Support workflow shown in the rightmost section of Fig. 2. The Quality Score triggers the reliability “Check,” while the Distortion Type guides the Adaptive Optimization, enabling a continuous Re-evaluation loop.

### Dual-stream semantic injection

Conventional IQA models typically rely on patch-based training, which often prioritizes local texture quality while neglecting global semantic content in salient regions. This limitation may lead to insufficient sensitivity to quality degradations occurring in semantically important areas, even when such degradations are perceptually critical for downstream tasks. To mitigate this limitation, a CLIP-based semantic injection mechanism is introduced. The distorted image is processed by a parameter-frozen pre-trained CLIP (ViT-B/16) image encoder to extract a global semantic vector. Freezing the CLIP encoder ensures that high-level semantic priors remain stable during training and prevents overfitting to dataset-specific distortion distributions. A semantic mapping network, consisting of two multi-layer perceptron (MLP) layers, projects this prior into the IQA feature space. Utilizing the multi-layer concatenated features $F \in {{\mathbb{R}}^{H \times W \times C}}$.

from the ViT backbone, a FiLM strategy generates channel-wise scaling ($\gamma$) and bias (β) factors using the semantic vector ${v_{{\mathrm{sem}}}}$:

1 $$\gamma ={\mathrm{ML}}{{\mathrm{P}}_\gamma }({v_{{\mathrm{sem}}}}),\;\;\;{\kern 1pt} \beta ={\mathrm{ML}}{{\mathrm{P}}_\beta }({v_{{\mathrm{sem}}}})$$

These modulation parameters enable semantic information to influence intermediate feature activations in a channel-adaptive manner. To ensure training stability and prevent semantic noise from disrupting pre-trained features during early phases, a Zero-Initialization strategy is applied to the output layers of ${\mathrm{ML}}{{\mathrm{P}}_\gamma }$ and${\mathrm{ML}}{{\mathrm{P}}_\beta }$, setting $\gamma =0$ and $\beta =0$ initially. This initialization strategy represents a distinct technical departure from conventional cross-modal attention pipelines that are typically computationally intensive. It enables the network to inherit generic semantic priors in a more controlled manner, without substantially disrupting the pre-trained local texture representations of the ViT backbone. As a result, the approach reduces the need for multi-stage fine-tuning while facilitating a lightweight form of semantic awareness.

The final semantically enhanced feature $F^{\prime}$ is computed as:

2 $${F^{\prime}_c}=(1+{\gamma _c}) \cdot {F_c}+{\beta _c}$$

where the subscript c denotes the channel index of the feature maps.

Consequently, the TAB module incorporates semantic context during channel attention computation, enabling adaptive weight adjustment that prioritizes semantically significant regions akin to human perception.

### Global-local feature interaction

Following semantic injection, features are propagated to the Multi-Dimension Interaction module. To robustly capture complementary perceptual dependencies, we adopt the hybrid attention architecture proposed in 10, which consists of two key units: TAB and SSTB.

The TAB computes a self-attention map across the channel dimension. In our framework, this process is explicitly reinforced by the previously injected semantic priors, enabling the TAB to capture global channel dependencies critical for perceptual quality. Channel-wise interactions are particularly important for modeling correlated degradation patterns that span multiple feature dimensions, such as blur or compression artifacts affecting structural and texture cues simultaneously.

Subsequently, the SSTB enhances spatial interactions among local image patches. By operating on localized windows with scale-aware attention, the SSTB strengthens sensitivity to spatially variant distortions while preserving contextual coherence across neighboring regions. By integrating these blocks into the proposed dual-stream architecture, the module yields a high-level feature representation that encodes not only local texture distortion and global channel correlations but also the injected semantic context. This sequential integration preserves both semantic modulation and fine-grained spatial structure within a unified feature embedding.

### Distortion diagnosis branch

To endow the intelligent software system with interpretability and fault diagnosis capabilities, a lightweight parallel Distortion Diagnosis Branch is incorporated. Conventional IQA models typically output only scalar scores without explanatory information. To address this limitation, the proposed diagnostic branch is designed to explicitly identify the specific distortion type responsible for quality degradation. The high-level features ${F_{{\mathrm{out}}}}$ from the SSTB undergo Global Average Pooling (GAP), followed by a classification head to predict the distortion probability distribution:

3 $${P_{{\mathrm{diag}}}}={\mathrm{Softmax}}({\mathrm{ML}}{{\mathrm{P}}_{{\mathrm{head}}}}({\mathrm{GAP}}({F_{{\mathrm{out}}}})))$$

This output provides actionable evidence for downstream adaptive systems—such as triggering specific sharpening algorithms upon detecting blur—thereby facilitating a transition from passive evaluation to adaptive optimization.

### Multi-task prediction and joint optimization

The primary quality scoring task adopts a dual-branch prediction structure, comprising a Scoring Branch and a Weighting Branch. The final perceptual quality score is derived via weighted summation:

4 $${S_{{\mathrm{pred}}}}=\frac{{\sum\limits_{{i=1}}^{N} {{w_i}} {s_i}}}{{\sum\limits_{{i=1}}^{N} {{w_i}} }}$$

Here, ${s_i}$ and ${w_i}$ denote the predicted score and local weight of the i-th image patch, respectively.

To simultaneously optimize quality prediction accuracy and distortion diagnosis, a multi-task joint loss function is employed for joint network optimization:

5 $${\mathcal{L}_{{\mathrm{total}}}}={\mathcal{L}_{{\mathrm{score}}}}+\lambda {\mathcal{L}_{{\mathrm{diag}}}}$$

where ${\mathcal{L}_{{\mathrm{score}}}}$ represents the Mean Squared Error (MSE) loss for quality regression, and ${\mathcal{L}_{{\mathrm{diag}}}}$ denotes the Cross-Entropy loss for distortion classification. The hyperparameter $\lambda$ balances the task weights. This joint optimization enables the diagnosis branch to provide auxiliary supervision to the shared feature extractor, thereby enhancing model robustness against complex distortions. Beyond merely providing an additional diagnostic label, the cross-entropy loss from this branch acts as a critical structural regularizer during backpropagation. It explicitly forces the shared high-dimensional feature manifold to disentangle complex degradation patterns into linearly separable sub-spaces. This mechanism directly addresses the feature coupling bottleneck inherent in pure scalar regression BIQA frameworks.

## Experiments

### Experimental setup

#### Datasets

To systematically assess the generalization capability and diagnostic reliability of the proposed model, four widely adopted IQA benchmark datasets were selected. As summarized in Table 1, these datasets exhibit strong complementarity in terms of sample scale, content diversity, and distortion distributions, thereby constituting a comprehensive evaluation suite that spans from controlled perceptual conditions to more complex and realistic scenarios.

**Table 1:** Experimental dataset statistics.

Dataset name	Year	No. of ref. images	No. of dist. images	Subjective score range
LIVE	2006	29	779	DMOS [0, 100]
CSIQ	2010	30	866	DMOS [0, 1]
TID2013	2015	25	3000	MOS [0,9]
KADID-10k	2019	81	10,125	[0, 5]

Specifically, LIVE and CSIQ are commonly used small-scale benchmarks, comprising 779 and 866 distorted images with five and six conventional distortion types, respectively. TID2013 contains 3000 images annotated with 24 fine-grained distortion categories, and its high diversity provides a suitable testbed for examining the model’s diagnostic sensitivity and robustness under complex degradation patterns. In addition, KADID-10k is a large-scale dataset including 10,125 distorted images across 25 distortion types, which is employed to evaluate the scalability of the framework and the robustness of its learned feature representations under data-intensive conditions.

**Fig. 3:**
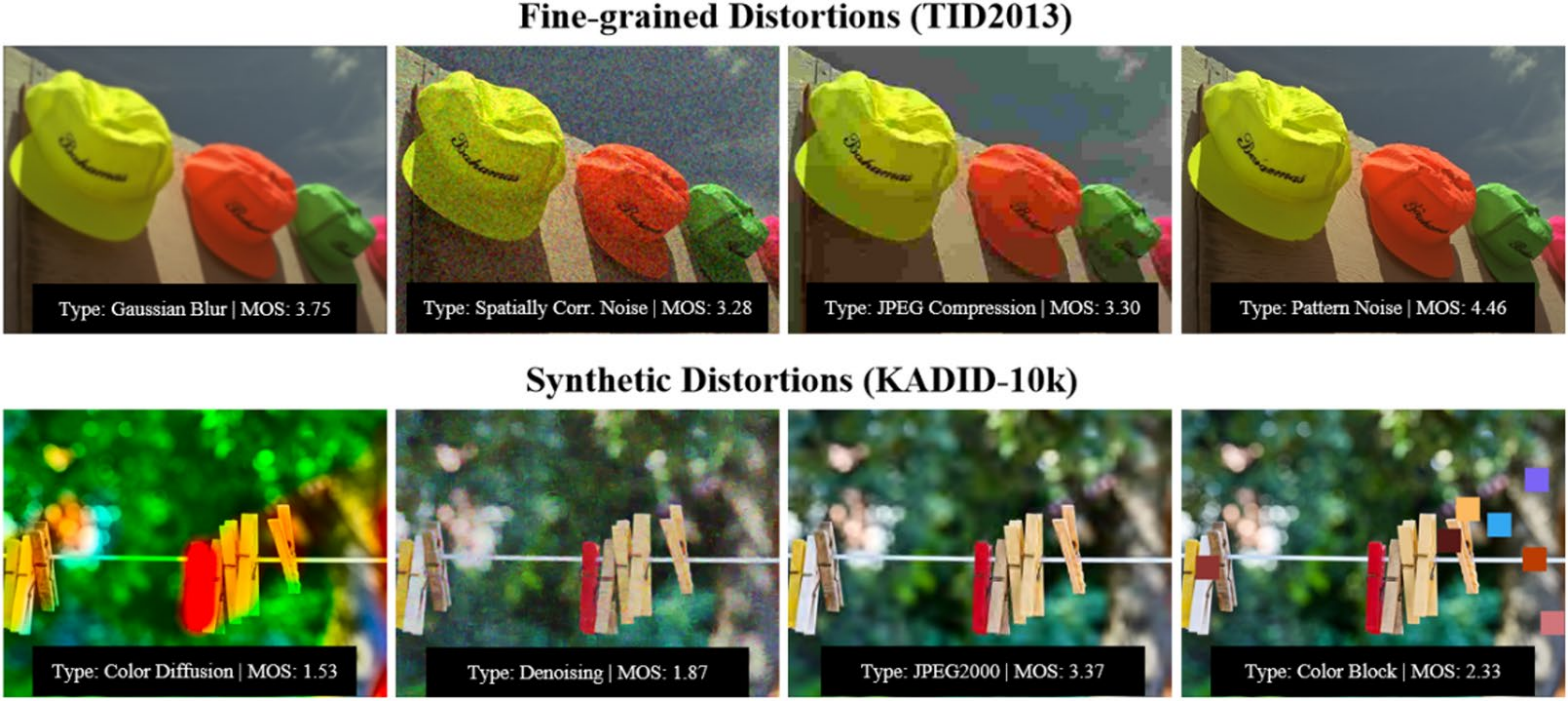
Visual diversity and perceptual challenges across benchmark datasets.

To further illustrate the perceptual complexity inherent in real-world distortions and to motivate the necessity of incorporating an explicit distortion diagnosis mechanism, representative samples from these benchmarks are presented in Fig. 3. The examples demonstrate a wide range of degradation patterns with notable inter-class visual ambiguity. For example, Color Diffusion artifacts in KADID-10k may exhibit visual characteristics similar to motion blur. In addition, denoising processes often introduce specific smoothing effects. Although perceptually distinct, these effects can be easily confused with conventional blur distortions. In contrast, synthetic transmission errors such as Color Block produce discrete digital artifacts that differ substantially from stochastic degradations such as Pattern Noise. These observations suggest that scalar quality scores alone may be insufficient to fully capture the underlying degradation characteristics, thereby motivating the introduction of an explicit diagnostic branch for more accurate distortion attribution.

#### Evaluation metrics

The Pearson Linear Correlation Coefficient (PLCC) and Spearman’s Rank Order Correlation Coefficient (SRCC) are adopted as evaluation metrics. Higher PLCC and SRCC values indicate superior performance in prediction accuracy, monotonicity, and consistency.

1. PLCC.

Measures the linear correlation between two variables. In IQA, it reflects the linear consistency between objective evaluation scores and subjective scores; a higher value indicates stronger linear correlation. The formula is as follows:

6 $${\mathrm{PLCC}}=\frac{{\sum\limits_{{i=1}}^{N} {({s_i} - \bar {s})} ({p_i} - \bar {p})}}{{\sqrt {\sum\limits_{{i=1}}^{N} {{{({s_i} - \bar {s})}^2}} } \sqrt {\sum\limits_{{i=1}}^{N} {{{({p_i} - \bar {p})}^2}} } }}$$

where *N* represents the number of test images, ${s_i}$ and ${p_i}$ represent the subjective score and predicted score of the *i*-th image, respectively, and $\bar {s}$ and $\bar {p}$ are their corresponding mean values.

2. SRCC.

Measures the monotonic correlation between two variables. In IQA, it primarily evaluates the consistency of the objective method in ranking image quality. A higher SRCC value indicates higher accuracy in quality ranking. The formula is as follows:

7 $$\rho = 1 - \frac{6 \sum d_i^2}{n(n^2 - 1)}$$

where $d_{i}^{{}}$ represents the difference in rank between the *i*-th data pairs, and *n* represents the total number of observed samples.

#### Implementation details

Experiments were conducted using PyTorch 2.5.1 on an NVIDIA RTX 4090. Data preprocessing included random cropping to a resolution of 224 × 224 and horizontal flipping with a probability of $p=0.7$ to enhance sample diversity. The model adopts an ImageNet-21k pre-trained ViT-B/8 backbone with a feature dimensionality of 768. The SSTB module is configured with a window size of 4, 4 attention heads, and a scaling factor of 0.80.

An 8:2 split was employed to divide the dataset into training and testing subsets. The partitioning was performed at the level of reference images to mitigate potential content overlap between the two subsets. For baseline comparisons, the reported results are averaged over five independent runs to assess generalization robustness. To further characterize statistical reliability, performance bounds are presented in terms of 95% confidence intervals (CIs). Subsequent ablation and zero-shot analyses are conducted under a fixed random seed, serving as a controlled experimental setting to isolate the effects of architectural modifications.

Model training utilized the Adam optimizer with a batch size of 8 and a weight decay of $1 \times {10^{ - 5}}$. The learning rate was scheduled using cosine annealing, where the maximum number of iterations ${T_{max}}$ was set equal to the total number of training epochs, and the minimum learning rate ${\eta _{min}}$ was set to 0. Hyperparameter settings were adjusted according to dataset scale, with initial learning rates of $1 \times {10^{ - 5}}$ for KADID-10k, $5 \times {10^{ - 6}}$ for TID2013, and $1 \times {10^{ - 6}}$ for smaller datasets such as LIVE and CSIQ. In addition, the diagnosis loss weight was set to $\lambda =0.5$. During inference, a five-point cropping strategy was adopted to obtain more stable performance estimates.

### Performance on standard benchmarks

To rigorously assess the effectiveness of the proposed semantic-aware BIQA framework, a systematic comparison is conducted against a set of representative state-of-the-art methods on four widely adopted benchmark datasets, namely LIVE, CSIQ, TID2013, and KADID-10k. As detailed in the implementation protocol, to mitigate the influence of stochastic factors and variations in data partitioning, all reported results are obtained by averaging the SRCC and PLCC values over five independent random splits. The quantitative results are summarized in Table 2, where “–” denotes that the corresponding results are unavailable due to the absence of original reports or publicly accessible implementations. From these results, it can be observed that the proposed method exhibits consistently stable and competitive performance across all considered benchmarks. Notably, its relative advantages become more apparent on datasets characterized by complex and diverse distortion types, suggesting its robustness in handling heterogeneous degradation scenarios.

On the TID2013 dataset, which includes 24 diverse and perceptually challenging distortion types, the proposed model achieves a mean SRCC of 0.9509 and a PLCC of 0.9552. To assess statistical significance relative to the state-of-the-art baseline MANIQA, a conservative one-sample *t*-test is conducted, defined as:

8 $$t=\frac{{\bar {x} - \mu }}{{s/\sqrt n }}$$

where $\bar {x}$ is the sample mean, *s* the standard deviation, *n* the number of trials, and $\mu$ the fixed baseline reference. The resulting *p*-values are 0.0183 for SRCC and 0.0020 for PLCC. As both are below 0.05, the results suggest that explicitly modeling distortion characteristics through the diagnosis branch contributes to improved performance in fine-grained degradation scenarios, where representation-centric methods may show limitations.

On the large-scale KADID-10k dataset, the proposed method achieves a mean SRCC of 0.9408 and a PLCC of 0.9435, outperforming recent single-stream vision–language and Transformer-based approaches. Given the scale and diversity of KADID-10k, this indicates that the dual-stream architecture, integrating semantic context and distortion diagnosis, provides strong scalability and representation capability.

For the smaller LIVE and CSIQ datasets, the proposed method maintains competitive accuracy. Although minor numerical variations are observed, with the baseline slightly outperforming on LIVE and the proposed method achieving higher mean scores on CSIQ (SRCC of 0.9640), statistical testing yields *p*-values above 0.05, such as $p=0.4769$ for LIVE SRCC and $p=0.5778$ for CSIQ SRCC. This indicates that the differences are not statistically significant and is consistent with the design objective of preserving comparable perceptual accuracy while providing additional diagnostic interpretability.

**Fig. 4:**
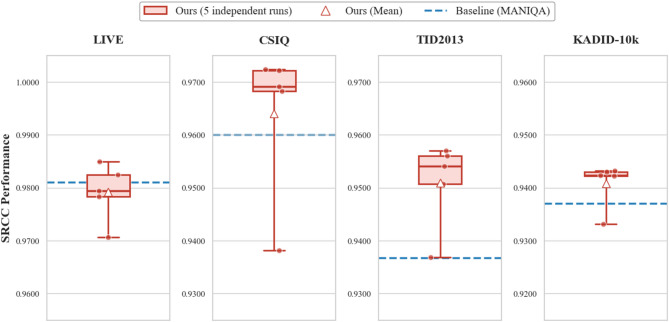
Statistical performance distribution (SRCC) of the proposed framework across five independent random trials.

To further illustrate stability, Fig. 4 presents the SRCC distributions across the five trials, with dashed lines indicating baseline performance. The relatively narrow variance ranges and the tight 95% confidence intervals, spanning from $\pm 0.0014$ to $\pm 0.0181$, indicate stable behavior across different data splits, suggesting that the learned representations are not overly sensitive to specific data configurations. The distribution patterns are also consistent with the statistical analysis, showing improved performance on TID2013 and comparable accuracy on other datasets.

In summary, the results on standard benchmarks indicate that the proposed semantic-aware and diagnostic-driven framework achieves competitive performance, with numerical, statistical, and distributional evidence supporting its stability and reliability as a perceptual monitoring component in adaptive intelligent systems.

**Table 2:** Performance comparison on standard benchmark datasets. Our results are Mean $\pm$ 95% CI over five splits.

	LIVE		CSIQ		TID2013		KADID-10 K	
	SRCC	PLCC	SRCC	PLCC	SRCC	PLCC	SRCC	PLCC
MEON^31^	0.9535	0.9496	0.8627	0.8515	0.8232	0.8068	0.6853	0.5972
WaDIQaM^32^	0.9540	0.9586	0.8426	0.8509	0.8545	0.8344	0.7462	0.7329
TIDQA^33^	0.9487	0.9635	0.8245	0.8369	0.8453	0.8564	0.8460	0.8506
MetaIQA^34^	0.9587	0.9581	0.8985	0.9072	0.8551	0.8678	0.7578	0.7709
P2I2M^35^	0.9588	0.9565	0.8987	0.9011	0.8608	0.8547	0.8363	0.8434
HyperIQ^36^	0.9611	0.9647	0.9219	0.9411	0.8390	0.8574	0.8485	0.8404
TRes^37^	0.9683	0.9677	0.9211	0.9413	0.8619	0.8818	0.9117	0.8536
MANIQA^10^	0.9811	0.9820	0.9601	0.9665	0.9368	0.9425	0.9371	0.9410
DEIQT^38^	0.9792	0.9815	0.9453	0.9628	0.9012	0.8910	0.8891	0.8867
LIQE^9^	0.9683	0.9502	0.9264	0.9386	–	–	0.9302	0.9305
LoDa^39^	0.9745	0.9786	–	–	0.8569	0.9007	0.9304	0.9356
Ours	0.9792 ± 0.0067	0.9801 ± 0.0063	0.9640 ± 0.0181	0.9703 ± 0.0153	0.9509 ± 0.0102	0.9552 ± 0.0048	0.9408 ± 0.0053	0.9435 ± 0.0014

### Generalization in zero-shot settings

The generalization capability and robustness of the proposed method were further evaluated using a zero-shot cross-dataset protocol. The model was trained on the large-scale KADID-10 K source domain and directly applied to heterogeneous target datasets, including LIVE, CSIQ, and TID2013, without parameter fine-tuning or domain adaptation, thereby approximating realistic deployment scenarios involving unseen data distributions and distortion types. As reported in Table 3, the proposed dual-stream architecture consistently outperforms the baseline in terms of ranking consistency across all target datasets. Specifically, it achieves SRCC values of 0.9286 and 0.8188 on LIVE and CSIQ, corresponding to improvements of 0.0138 and 0.0162, respectively, and further improves SRCC on the more challenging TID2013 dataset from 0.7148 to 0.7211. Notably, a divergence between SRCC and PLCC is observed on TID2013, which can be attributed to the domain discrepancy between the pre-training distribution of the CLIP vision encoder and the characteristics of the dataset. While CLIP is trained on large-scale natural image–text pairs and thus captures priors aligned with semantically meaningful real-world degradations such as blur or underexposure, TID2013 contains a substantial proportion of synthetically generated, mathematically defined distortions that are rarely encountered in natural visual environments. Consequently, although the proposed framework preserves relative perceptual ordering, as reflected by SRCC, this domain gap may limit its ability to accurately map such out-of-distribution distortions onto an absolute linear severity scale, thereby affecting PLCC. Nevertheless, the model maintains stable perceptual assessment performance on unseen content, supporting its applicability in open-world adaptive systems.

**Table 3:** Zero-shot cross-dataset evaluation results.

Testing set	Metric	Baseline (MANIQA)	Ours	Gain
LIVE	SRCC	0.9148	0.9286	+ 0.0138
	PLCC	0.8988	0.9131	+ 0.0143
CSIQ	SRCC	0.8026	0.8188	+ 0.0162
	PLCC	0.8304	0.8566	+ 0.0262
TID2013	SRCC	0.7148	0.7211	+ 0.0063
	PLCC	0.7328	0.7299	− 0.0029

### Data efficiency and learning robustness

**Fig. 5:**
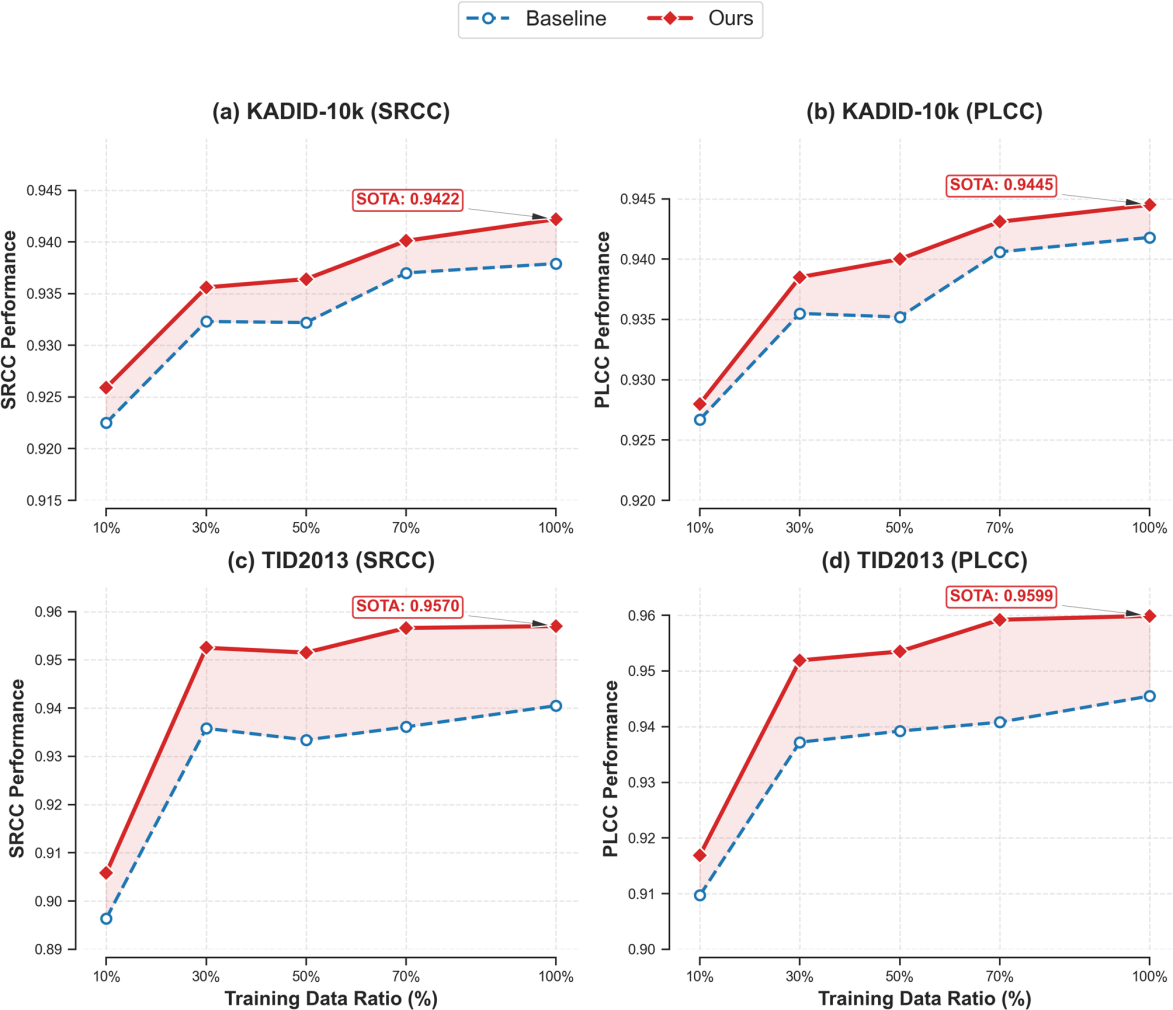
Data efficiency analysis on KADID-10k and TID2013.

To evaluate the deployment feasibility and data cost-effectiveness of the proposed method in software intelligence scenarios, experiments were conducted on the KADID-10k and TID2013 datasets with training data ratios ranging from 10% to 100%. As shown in Fig. 5, the proposed method consistently outperforms the baseline, demonstrating superior robustness particularly in data-scarce regimes essential for reducing manual annotation overhead. For instance, on the limited TID2013 dataset, the method maintains a reliable SRCC of 0.9058 using only 10% of the data, whereas the baseline degrades to 0.8964. Furthermore, the proposed approach exhibits rapid convergence, achieving an SRCC of 0.9525 with merely 30% of the training samples—significantly surpassing the baseline and approximating the optimal performance. These results indicate that the method can effectively model quality features with minimal data dependence, offering a label-efficient solution for the practical deployment of automated visual monitoring systems.

### Distortion diagnosis capability

To evaluate the effectiveness of the proposed framework in distortion diagnosis for adaptive software systems, the diagnostic branch is assessed through both quantitative metrics and visualization-based analyses. Top-1 classification accuracy is adopted to measure distortion identification performance across datasets with varying scales and distortion complexities, as summarized in Table 4.

With the incorporation of CLIP semantic priors and multi-task joint optimization, the model demonstrates stable diagnostic performance across all evaluated benchmarks under standard intra-dataset train–test splits. Specifically, it achieves an accuracy of 96.17% on TID2013, which contains fine-grained and diverse distortion categories, while maintaining accuracies of 91.48% and 95.83% on the large-scale KADID-10k and CSIQ datasets, respectively. Due to its limited sample size, the LIVE dataset does not provide sufficient statistical support for reliable fine-grained diagnosis and is therefore excluded from this quantitative evaluation.

**Table 4:** Top-1 diagnostic accuracy on standard benchmark datasets.

Dataset	Number of distortion types	Diagnostic accuracy
KADID-10k	25	91.48%
TID2013	24	96.17%
CSIQ	6	95.83%

**Fig. 6:**
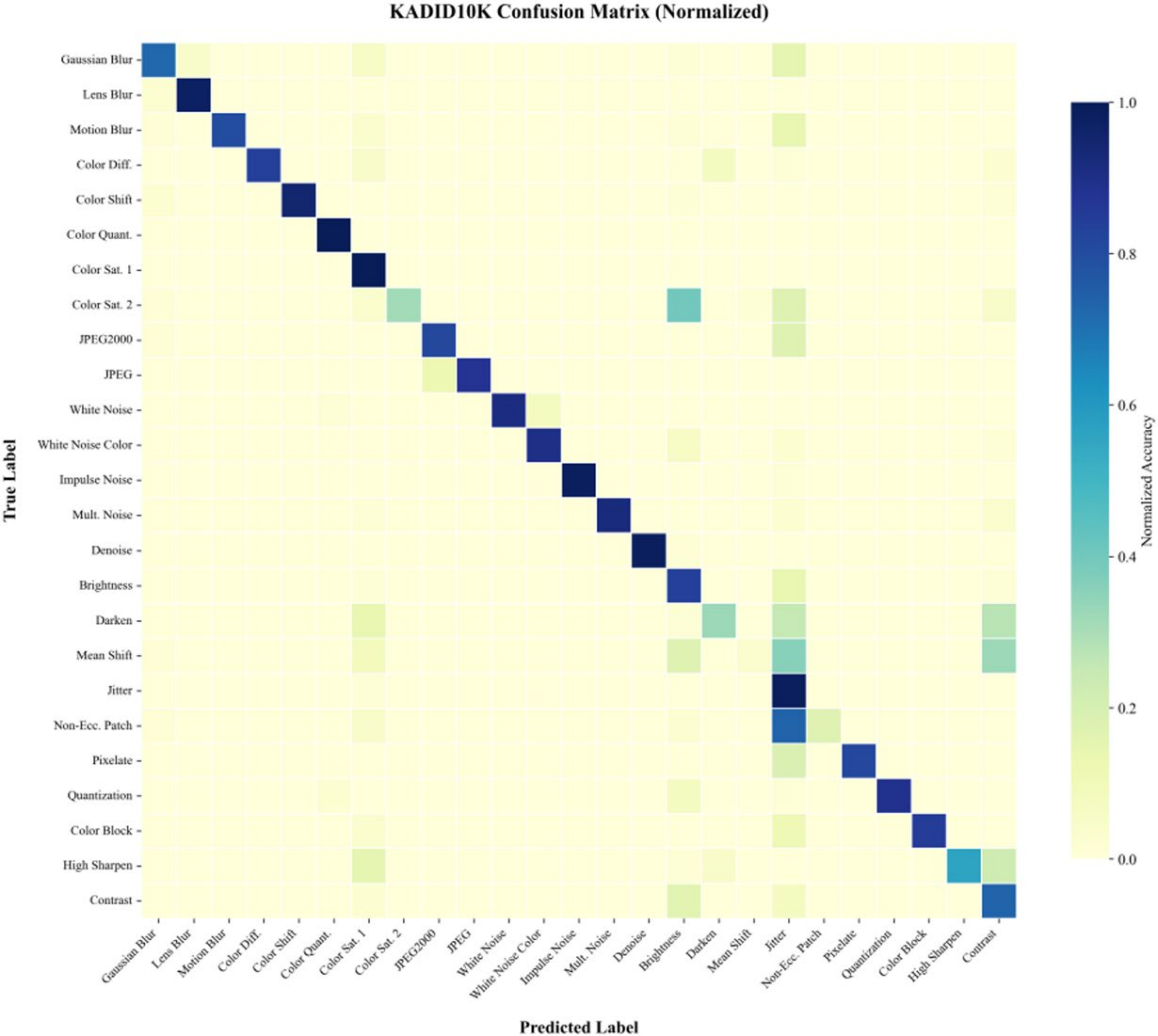
Normalized confusion matrix on KADID-10k, illustrating the discriminative capability of the diagnostic branch across different distortion types.

To further examine category-level reliability, a normalized confusion matrix on KADID-10k is presented in Fig. 6. The result shows clear diagonal dominance, indicating effective separation among distortion types. Distortions with distinctive visual characteristics, such as Gaussian noise and compression artifacts, are identified with relatively high precision, while residual confusions are mainly concentrated among perceptually similar categories. This pattern suggests that the diagnostic branch captures structured distortion representations rather than relying primarily on label memorization.

To assess generalization beyond dataset-specific characteristics, a cross-dataset diagnostic experiment is conducted. Considering domain discrepancies introduced by different degradation synthesis processes, particularly in earlier benchmarks, the evaluation is performed at the super-class level, including blur, noise, and compression categories. A model trained exclusively on the KADID-10k source domain is directly applied in a zero-shot manner to the unseen TID2013 and LIVE datasets. The resulting accuracies are 82.00% on TID2013 and 90.91% on LIVE, indicating a reasonable degree of cross-domain generalization while also reflecting the challenges posed by domain shifts in diverse operational settings. These observations further motivate the use of closed-loop mechanisms to maintain system reliability under previously unseen degradations.

To complement the cross-dataset evaluation with a more fine-grained analysis, per-class precision, recall, and F1-score are computed across all 25 distortion categories on KADID-10k, with detailed results provided in the Supplementary Material. The model achieves a macro-average F1-score of 0.9428. Misclassifications are primarily confined to visually overlapping or semantically similar artifacts, such as slight illumination variations versus contrast shifts, which can be attributed to their similar frequency-domain characteristics.

To further examine the impact of such misclassifications on quality prediction, the prediction error is quantified on the KADID-10k test set. For correctly diagnosed samples, the Mean Absolute Error (MAE) of the predicted quality score is 0.186, while for misclassified samples it increases to 0.303, indicating a moderate reduction in accuracy. Considering the 1-to-5 MOS scale, this result suggests that the shared feature representations within the ViT backbone remain robust. Even when perceptually similar distortions are confused, the model largely preserves degradation severity, thereby limiting error propagation to the final quality prediction and supporting overall system reliability.

Overall, these results indicate that the proposed framework, particularly the diagnostic branch within the dual-stream architecture, provides reliable and interpretable distortion diagnosis by promoting the separation of distortion-related features from semantic content. This capability supports its use as a functional component for downstream adaptive processing, including distortion-aware quality assessment and targeted restoration.

### Ablation study

Ablation experiments were conducted on the KADID-10 K and TID2013 datasets to examine the contribution of the proposed components. To ensure a fair comparison and isolate the effects of architectural modifications, all model variants, including the baseline, were evaluated under the identical fixed random seed. Model variants were constructed by integrating the semantic injection and distortion diagnosis branches, as detailed in Table 5. The results demonstrate the complementary nature of the two modules. Specifically, the variant equipped with semantic priors (w/ Semantic) improved over the baseline, increasing the SRCC on TID2013 from 0.9405 to 0.9527. This suggests that incorporating high-level semantic priors assists in perceptual evaluation, especially when local texture information is limited.

**Table 5:** Ablation test results of key components evaluated under an identical fixed random seed.

Model variant	KADID-10 K		TID2013	
	SRCC	PLCC	SRCC	PLCC
Baseline	0.9379	0.9428	0.9405	0.9455
w/ Semantic	0.9390	0.9430	0.9527	0.9553
w/ Diagnosis	0.9354	0.9384	0.9601	0.9628
Ours	0.9422	0.9445	0.9570	0.9599

Regarding the distortion diagnosis branch (w/ Diagnosis), performance varied when implemented without semantic guidance. While it showed effectiveness on TID2013, a slight decline was observed on KADID-10 K, implying that auxiliary tasks might introduce optimization trade-offs in the absence of semantic context. Ultimately, the proposed framework (Ours) achieved the highest overall performance on KADID-10 K with an SRCC of 0.9422, indicating that the joint modeling of semantic and distortion features provides a balanced and generalizable representation for diverse image quality assessment tasks.

### Visual explanations

**Fig. 7:**
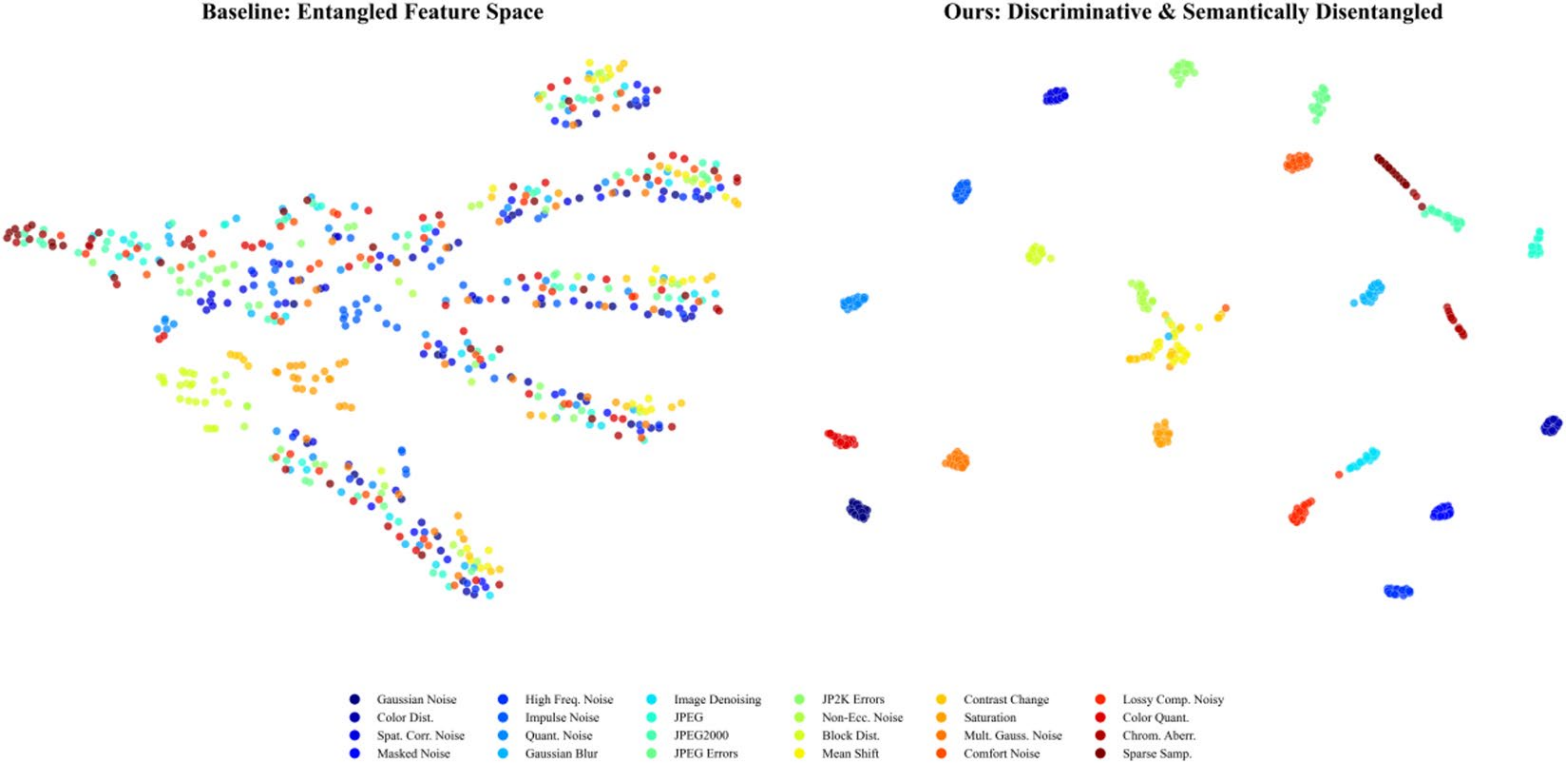
Visualization and comparison of t-SNE feature manifolds on the TID2013 dataset.

Qualitative validation of perceptual discrimination was performed using t-SNE visualization of high-dimensional feature embeddings from the TID2013 dataset, as depicted in Fig. 7. In contrast to the baseline MANIQA model, the proposed dual-stream architecture demonstrates superior feature disentanglement. Driven by semantic priors and diagnostic supervision, the network successfully maps 24 fine-grained distortion patterns into distinct, compact, and independent clusters. This structured feature distribution indicates the acquisition of distortion-specific representations with explicit physical significance rather than simple statistical fitting, providing a robust foundation for the interpretability of adaptive intelligent systems. Consequently, the ability to produce a semantically disentangled and physically interpretable feature space within a single forward pass can be regarded as a central methodological contribution of the proposed dual-stream architecture, distinguishing it from approaches based on straightforward module stacking.

To examine the influence of semantic guidance on spatial perceptual focus, we performed a qualitative analysis of attention maps generated by Grad-CAM on representative samples from the KADID-10k dataset. For globally distributed degradations, such as Gaussian blur and white noise, the baseline model produces widely dispersed activations across the image, reflecting its sensitivity to low-level texture statistics. Although this behavior is consistent with representation-driven architectures, it may limit the model’s ability to selectively emphasize perceptually salient regions. In contrast, the proposed framework, guided by CLIP-derived semantic priors, yields comparatively more spatially concentrated responses over semantically meaningful foreground areas. This observation suggests a closer alignment with human visual attention patterns and indicates that semantic modulation can encourage the model to allocate greater emphasis to content-relevant regions, thereby potentially facilitating more content-aware quality assessment in complex visual scenarios.

### Hard-sample and failure-case analysis

**Fig. 8:**
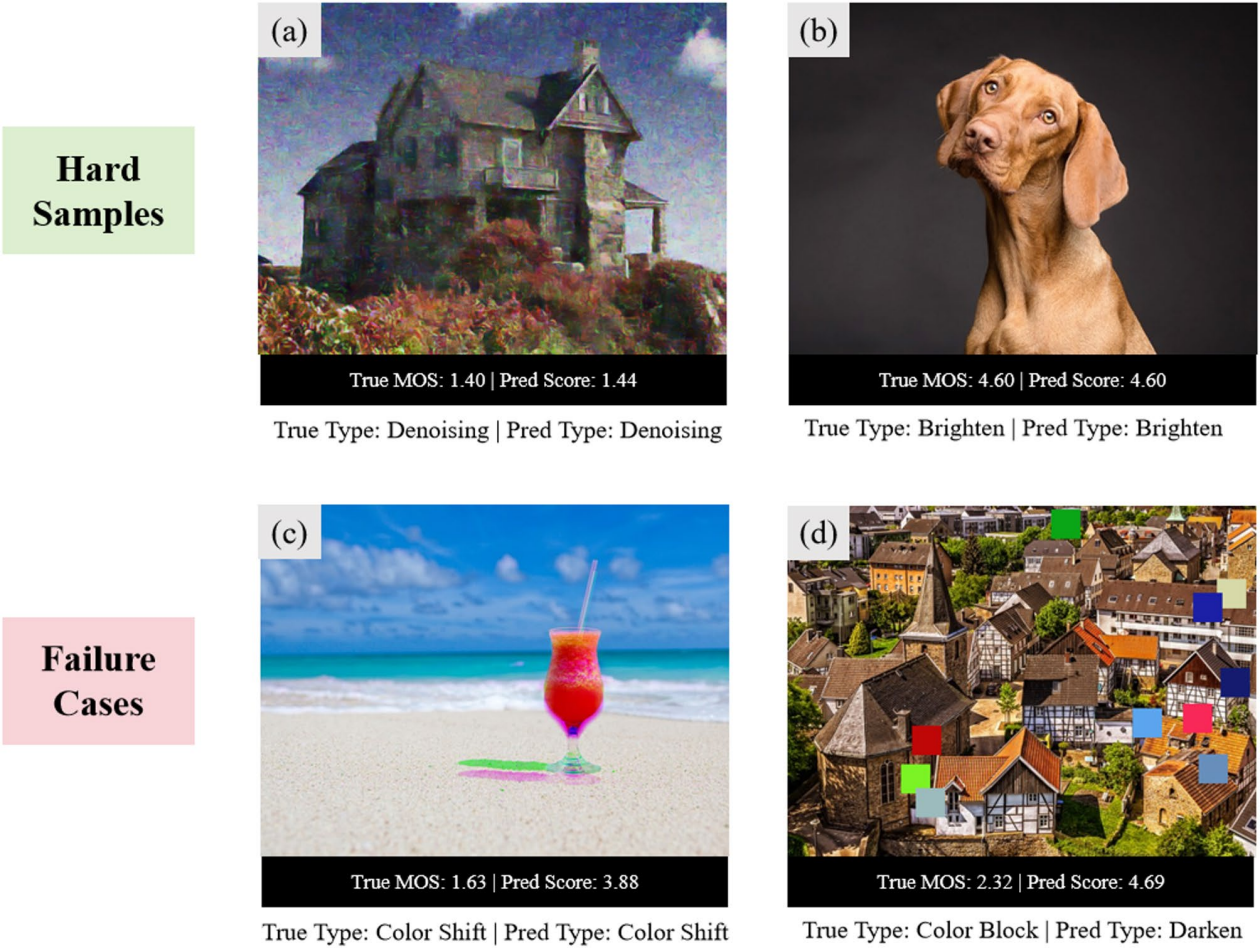
Qualitative analysis of hard samples and failure cases on the KADID-10k dataset.

While the proposed framework demonstrates competitive overall performance, examining its behavior on challenging samples and failure cases provides additional insight into the contributions and operational boundaries of the semantic guidance and diagnosis branches. Representative examples from the KADID-10k dataset are shown in Fig. 8.

As illustrated in Fig. 8a,b, the framework performs effectively on perceptually challenging samples. In Fig. 8a, the image is affected by pronounced denoising artifacts. Conventional approaches often encounter difficulty in distinguishing heavy denoising from structural blur due to their similar smoothing characteristics. In contrast, the proposed diagnosis branch is able to disentangle these factors, correctly identifying the distortion as “Denoising” and producing a prediction that closely aligns with the corresponding subjective score. Figure 8b further highlights the role of semantic guidance. Despite the presence of complex background distortions, the model attends to semantically salient regions associated with the primary subject, yielding a prediction consistent with human perceptual evaluation.

In contrast, Fig. 8c,d present representative failure cases that reveal the limitations of the proposed method. In Fig. 8c, the observed performance degradation is associated with the disruption of natural visual priors. The Color Shift distortion significantly alters the inherent color distribution of the scene, producing unnatural representations of elements such as the sky and ocean. As the frozen CLIP encoder relies on learned natural visual semantics, such deviations may adversely affect feature extraction. Although the diagnosis branch correctly identifies the distortion type, the corrupted semantic cues may interfere with the subsequent quality regression, resulting in an overestimation of the predicted score. To address this limitation, future work may incorporate uncertainty estimation to quantify prediction reliability under severe semantic deviations. Identifying out-of-distribution samples would enable adaptive confidence calibration or the flagging of potentially unreliable predictions. In addition, more advanced multimodal fusion strategies could be explored, for example by introducing textual prompts as complementary semantic anchors, thereby enabling cross-modal verification and supporting a more stable perceptual focus under pronounced color distortions. Figure 8d illustrates a more extreme degradation scenario. When high-frequency structural information is substantially impaired, perceptually distinct distortions become increasingly difficult to distinguish. Under such conditions, the single-frame diagnosis mechanism exhibits ambiguity, leading to the misclassification of a Color Block distortion as Darken, which in turn degrades the final prediction accuracy. Collectively, these observations delineate the operational boundaries of the current visually-driven framework, further reaffirming the necessity of developing the aforementioned uncertainty-aware and cross-modal calibration mechanisms for future robust deployments.

### Computational efficiency and operational feasibility analysis

To quantitatively assess the operational feasibility of the proposed framework for adaptive intelligent systems, computational efficiency was evaluated on an NVIDIA RTX 4090 with a batch size of one. As reported in Table 6, the proposed model achieves a throughput of 65.00 frames per second (FPS) with an average latency of 15.42 ms. This reflects a modest latency increase of approximately 2.0 ms compared with the representative baseline (ViT-B + Swin). Despite this increment, the model maintains performance well above the commonly adopted real-time threshold of 30 FPS, thereby preserving adequate temporal capacity for downstream decision-support processes.

Although the total parameter count increases to 290.54 million, a considerable portion of these parameters originates from the CLIP vision encoder, which remains frozen during training and thus does not introduce additional optimization overhead. Furthermore, the overall computational complexity, measured in floating-point operations (FLOPs), increases by only 8.5%, indicating that the incorporation of semantic reasoning introduces a relatively limited computational burden.

More importantly, Table 6 highlights a favorable balance between computational efficiency and functional enhancement. While the baseline model operates slightly faster, it is limited to predicting a single scalar quality score with comparatively lower accuracy, achieving an SRCC of 0.9386 on TID2013. In contrast, the proposed Multi-Task Prediction Head not only improves perceptual accuracy, reaching an SRCC of 0.9509, but also extends the output capability to jointly produce continuous quality scores and explicit distortion classifications within a single forward pass. This unified design facilitates computational reuse and provides a principled justification for the minor latency increase, supporting its suitability as an effective real-time diagnostic component in decision-support systems.

**Table 6:** Inference efficiency comparison.

Model	Backbone	Params (M)	Latency (ms)	FPS (images/s)	SRCC (TID2013)	Output capability
Baseline	ViT-B + Swin	135.75	13.42	74.53	0.9386	Scalar score only
Ours	ViT-B + CLIP + Diagnosis	290.54	15.42	65.00	0.9509	Score + distortion type

### Discussion: implications for adaptive intelligent systems

To evaluate its utility as an embedded perception component, the proposed framework is integrated into a closed-loop image triage simulation. As illustrated in the closed-loop decision support module of Fig. 2, this design reformulates BIQA from a passive scoring task into an interpretable perception module embedded within the processing pipeline. Unlike conventional offline evaluators, the system performs real-time analysis of operational visual inputs. Inputs that satisfy predefined reliability thresholds are forwarded to downstream modules to maintain computational efficiency, whereas degraded inputs activate an adaptive optimization workflow.

**Fig. 9:**
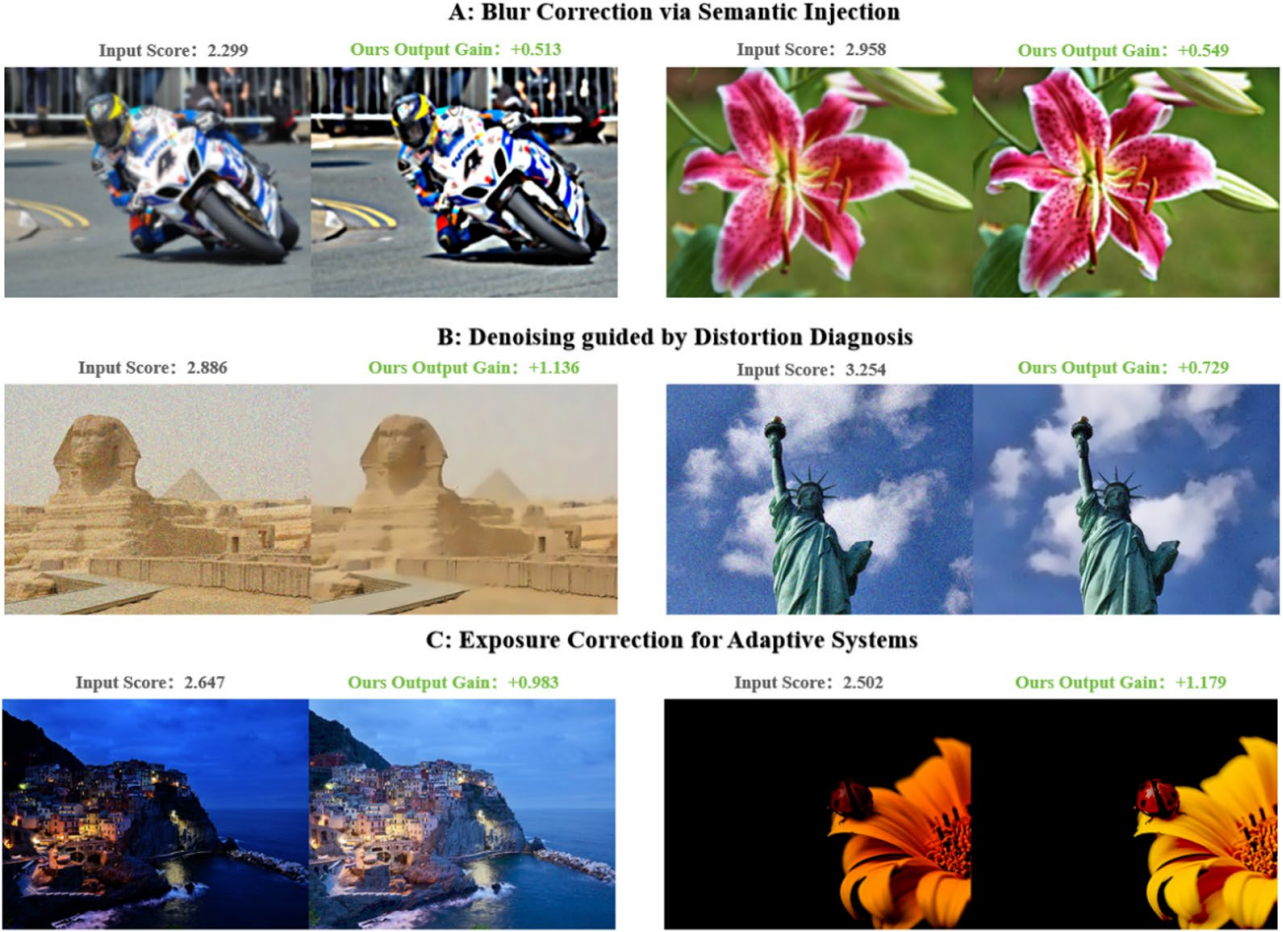
Verification of the closed-loop perception reliability mechanism, demonstrating diagnosis-guided restoration and the re-evaluation cycle.

Guided by the distortion diagnosis branch, the system applies targeted restoration strategies rather than uniform processing. As illustrated in Fig. 9, different degradation types are associated with corresponding correction routines. For instance, motion blur identified in the racing car scenario triggers a deblurring process, while noise detected in the Sphinx example activates a dedicated denoising operation. These diagnostic outputs provide structured and actionable cues for subsequent processing modules. Following restoration, the outputs undergo a re-evaluation stage, allowing the system to assess perceptual improvement prior to final acceptance. The simulation results suggest that the model can distinguish among motion blur, high-frequency noise, and underexposure, and accordingly select suitable correction strategies that lead to observable perceptual improvements.

This mechanism introduces an operational assurance layer at the perception level. By detecting and mitigating sensory degradation, it reduces the likelihood that unreliable inputs propagate into downstream reasoning processes, a capability that is particularly relevant in dynamic environments. This form of assurance complements higher-level verification strategies by providing structured and interpretable evidence of input quality, thereby supporting the stability of the overall decision-making process.

It is important to delineate the scope and boundaries of this work with precision. While the proposed methodology establishes a perception-level loop that integrates monitoring, diagnosis, and simulated restoration, it should be regarded as an algorithmic foundation rather than a fully developed end-to-end system deployment. The current experimental results primarily validate the effectiveness of the diagnostic routing mechanism. Extending this conceptual closed-loop framework to real-world operational settings requires addressing several system-level considerations. The serialized structure of the diagnosis, restoration, and re-evaluation pipeline may introduce non-negligible feedback latency, which could limit efficiency in high-throughput or real-time visual processing scenarios. In addition, perceptual degradations in practical environments often occur in mixed and concurrent forms, such as the coexistence of low-light noise and motion blur, rather than as isolated distortions. Under such conditions, relying on a single dominant diagnosis to activate a specialized restoration module may be insufficient and could potentially lead to suboptimal outcomes or secondary artifacts. Accordingly, improving the pipeline to support lower-latency feedback and developing multi-label diagnostic routing strategies for mixed-distortion restoration represent important directions for future work.

To explicitly quantify the end-to-end perceptual benefits of the proposed closed-loop architecture, a comparative pipeline analysis was conducted based on the representative scenarios shown in Fig. 9. In this analysis, the diagnosis-guided adaptive routing approach was compared with two conventional strategies: a non-adaptive strategy, which uniformly applies a fixed denoising filter to all images, and a mismatched strategy, which employs an inappropriate restoration algorithm. The resulting quality gains, defined as the differences in perceptual scores before and after processing, are reported in Table 7.

**Table 7:** Quantitative comparison of different restoration strategies.

Input scenario	Non-adaptive (always denoise)	Mismatched	Ours (diagnosis-guided)
Blur (racing car)	− 0.271	− 0.296	+ 0.513
Noise (sphinx)	+ 0.955	− 0.767	+ 0.955
Exposure (flower)	+ 0.001	− 0.138	+ 1.179

As indicated in Table 7, both non-adaptive and mismatched restoration strategies often lead to negative quality gains, suggesting that the application of unsuitable processing operations, such as sharpening a noise-contaminated image or denoising a blurred image, may introduce noticeable secondary degradations. In contrast, the proposed diagnosis-guided framework consistently yields higher positive gains across the evaluated scenarios, as it facilitates the selection and activation of an appropriate restoration mechanism. These quantitative results suggest that the diagnostic branch functions not merely as an auxiliary labeling component, but as an important operational module that contributes to the overall reliability and stability of the end-to-end system.

## Conclusion

This work addresses the challenge of perception reliability in large-scale visual communication and automated processing environments by proposing an interpretable framework that unifies semantic-aware quality assessment with explicit distortion diagnosis. Rather than focusing solely on architectural modifications, the approach reconceptualizes blind image quality assessment as a perception component capable of characterizing degradation patterns and supporting system-level awareness. Experimental results on standard benchmarks, including TID2013 and KADID-10k, indicate competitive performance and consistent cross-domain generalization, supporting the effectiveness of the framework under diverse and complex distortion conditions. From a software engineering perspective, the framework can be viewed as an interpretable decision-support mechanism for adaptive intelligent systems. It enables closed-loop monitoring of perceptual reliability and facilitates targeted restoration, thereby reducing the risk that unreliable inputs propagate through downstream perception–reasoning–decision pipelines. Future work will explore richer forms of perception-level feedback, including the integration of large multimodal models, to enhance the expressiveness of quality assessment outputs. By progressively extending numerical quality indicators toward semantically grounded representations, these efforts aim to improve the transparency, adaptability, and autonomy of next-generation intelligent perception systems.

## Supplementary information

Supplementary Information.

## Data Availability

The datasets generated and/or analysed during the current study are available in publicly accessible repositories. The specific data generated by the proposed model during the current study are available from the corresponding author on reasonable request.The source code and pre-trained inference models for the proposed framework are publicly available on GitHub at: https://github.com/eggajr001-rgb/Explainable-BIQA.
