# Supplementary material for: Explainable blind image quality assessment with closed-loop semantic guidance and distortion diagnosis
Source: Sci Rep. 2026 May 9;16:19350. doi: 10.1038/s41598-026-51187-6 (PMC13287672; doi:10.1038/s41598-026-51187-6)
Supplement: Supplementary file 1 — Supplementary Information. [file 41598_2026_51187_MOESM1_ESM.pdf]

Class ID	Distortion Type	Precision	Recall	F1-Score
01	Gaussian blur	0.8769	0.6706	0.7600
02	Lens blur	0.9605	0.8588	0.9068
03	Motion blur	0.8313	0.8118	0.8214
04	Color diffusion	1.0000	1.0000	1.0000
05	Color shift	0.9444	1.0000	0.9714
06	Color quantization	1.0000	1.0000	1.0000
07	Color saturation 1	1.0000	1.0000	1.0000
08	Color saturation 2	1.0000	0.8000	0.8889
09	JPEG2000	1.0000	0.9882	0.9941
10	JPEG	1.0000	1.0000	1.0000
11	White noise	1.0000	1.0000	1.0000
12	White noise color	0.9884	1.0000	0.9942
13	Impulse noise	1.0000	1.0000	1.0000
14	Multiplicative noise	1.0000	0.9882	0.9941
15	Denoise	1.0000	0.9882	0.9941
16	Brightness	1.0000	0.8941	0.9441
17	Darken	0.4775	1.0000	0.6464
18	Mean shift	1.0000	0.6824	0.8112
19	Jitter	0.9880	0.9647	0.9762
20	Non-eccentricity patch	0.9425	0.9647	0.9535
21	Pixelate	1.0000	0.9882	0.9941
22	Quantization	1.0000	1.0000	1.0000
23	Color block	0.9867	0.8706	0.9250
24	High sharpen	1.0000	1.0000	1.0000
25	Contrast change	1.0000	0.9882	0.9941
Avg	Macro Average	0.9599	0.9384	0.9428

Table S1. Per-class Precision, Recall, and F1-score of the diagnostic branch on KADID-10k.
